# To the Editors of the Medical and Physical Journal

**Published:** 1802-07

**Authors:** W. White

**Affiliations:** Bath


### *To the Editors of the Medical and Phyſical Journal.*

Gentlemen,

I Thought proper to ſend the ſubjoined caſe, as another inſtance of the efficacy of opiate friction, corroborative of Mr. Ward's ſtatements, although the caſe occurred long before his Remarks were publiſhed. I am, &c.

*Bath, June* 11, 1802. W. WHITE.

John Bulmer, aged 54, a ſervant of Col. G——, was viſited by Mr. Creaſer, ſurgeon, of this city, in April, 1800, on account of a ſevere ſtrain in the ankle joint. On the ſecond day after the accident a delirium ſucceeded, which rendered his admiſſion into the Infirmary neceſſary. When I ſaw him, he complained of a pain in his left ſide, attended with a troubleſome cough. His pulſe was 106, and the ſtroke hard; his tongue was clean, and his bowels open. The ankle was ſomewhat ſwelled and diſcoloured, but he did not complain of any pain in that part. Although he gave a tolerably clear account of himſelf, yet there appeared at the ſame time a conſiderable degree of wildneſs about him, and incoherency of ſpeech; his eyes were much ſuffuſed, and the pupil contracted. The antiphlogiſtic plan was adopted, and the blood taken from his arm put on an inflamed appearance. The delirium, however, increaſed to ſuch a degree that it became abſolutely neceſſary to confine him. He was cupped; the temporal artery was opened; bliſters were ſucceſſively applied to the back, and inſide of the legs, and ſinapiſms to his feet. Opiates were given him, and alſo digitalis pretty freely. Notwithſtanding all theſe means were made uſe of, he remained without any ſleep for five days, ſtill continuing highly delirious, at laſt refuſing every thing that was offered him, whether of food or medicine. In this deplorable ſituation it was determined to try the effects of opiate friction. One ſcruple of opium was diſſolved in half an ounce of ſpiritus ætheris vitriolici comp. half of which I rubbed on the inſide of his thigh, at 10 o'clock P. M. and directed the remainder to be uſed in the ſame manner, at 2 o'clock, A. M. The next morning there appeared no alteration whatever; but at two o'clock in the afternoon he was ſeized with a violent convulſive fit, which terminated in a profound ſleep of twelve hours continuance; when he awoke perfectly ſenſible. For two or three days he ſcarcely awoke but to take his food; but there was no return of delirium, and in a ſhort time he perfectly recovered.

*Continuation*

